# Supplementary material for: Decoupled oxidation process enabled by atomically dispersed copper electrodes for in-situ chemical water treatment
Source: Nat Commun. 2024 Feb 8;15:1186. doi: 10.1038/s41467-024-45481-y (PMC10853265; doi:10.1038/s41467-024-45481-y)
Supplement: Supplementary file 1 — Supplementary Information [file 41467_2024_45481_MOESM1_ESM.pdf]

Supplementary Information

Decoupled oxidation process enabled by atomically dispersed copper electrodes for in-situ chemical water treatment

Ziwei Yu[1], Xuming Jin[1], Yang Guo[1], Qian Liu[2], Wenyu Xiang[1], Shuai Zhou[1], Jiaying Wang[1], Dailin Yang[1],

Hao Bin Wu[2]* & Juan Wang[1]*

[1]*Institute of Environmental Health, College of Environmental & Resource Sciences, Zhejiang University, Hangzhou 310058, China.*
[2]*Institute for Composites Science Innovation (InCSI) and State Key Laboratory of Silicon Materials, School of Materials Science and Engineering, Zhejiang University, Hangzhou 310027, China.*

**Corresponding* authors. E-mail: wjuan@zju.edu.cn; hbwu@zju.edu.cn

This PDF file includes:

Supplementary Methods

Supplementary Figures 1 to 32

Supplementary Tables 1 to 7

Supplementary References

Supplementary Methods

Materials.

Sodium hydrogen carbonate (99.5%), calcium chloride anhydrous(96.0%), hydrochloric acid (36~38.0 wt%), boric acid (99.8%), ethylenediamine tetraacetate acid disodium salt dihydrate (99.0%), acetone (99.5%), ethyl alcohol (99.7%) and sodium chloride (99.5%) were ordered from Sinopharm Chemical Reagent Co., Ltd. Nafion solution (5.0 wt%), bisphenol A (99.0%), copper (Ⅱ) acetate monohydrate (99.0%), potassium ferricyanide (99.5%), *p*-nitrophenol (98.0%), peroxydisulfate (99.5%), 5,5-dimethyl-1-pyrroline-N-oxide (DMPO, 99.0%) and 2,2,6,6-tetramethylpiperidine (TEMP, 98.0%) were ordered from Aladdin Chemistry Co., Ltd. Dicyandiamide (99.0%), 1,3,5-benzenetricarboxylic acid (99.0%) and 2,4-dichlorophenol (99.0%) were ordered from J&K scientific Co., Ltd. Potassium iodide (99.0%), L-glutamic acid (99.0%), phenol (99.5%), *p*-chlorophenol (99.0%), copper nitrate (99.0%), sodium tetraborate (99.5%) and methanol (99.9%) were ordered from Macklin Biochemical Technology Co. Ltd. Deionized (DI) water with a resistivity of 18.2 MΩ cm^{-1} was produced via a Millipore Mili-Q IQ 7000. Commercial graphite felts with over 99.0% carbon content were purchased from Tianjin Carbon Plant. The proton exchange membrane (PEM) Nafion membrane N-117 was purchased from the Chamours Company. Multi-walled carbon nanotubes (95.0%) was purchased from Tanfeng Technology Incorporated. Upper Mississippi River NOM No.1R110N was purchased from Shanghai Qifa Experimental Reagent Co., LTD. All the chemicals and materials are directly used without special notification.

Characterization. Morphologies of the samples were observed via field emission scanning electron microscope (FE-SEM, SU8010, Hitachi, Japan), transmission electron microscope (TEM, JEM-1200EX, Japan). Atomic high-angle annular dark field scanning transmission electron microscopy (HAADF-STEM) characterization was conducted on a probe corrected transmission electron microscope (FEI Titan G^2 80-200 ChemiSTEM) equipped with an energy dispersive X-ray spectroscopy. Brunauer-Emmett-Teller (BET) specific surface area was obtained on a gas sorption analyzer (TriStar II, Quantachrome Instruments) at liquid nitrogen temperature. X-ray diffraction patterns were carried out on a powder X-ray diffractometer (Bruker D8 ADVACNCE, Bruker, Germany). The X-ray absorption spectra (XAS) at Cu K-edge were measured at BL07A1 of National Synchrotron Radiation Research Center, using a Si (111) double-crystal monochromator. Hybrid Quadrupole-TOF $LC^{-}MS/MS$ mass spectrometer, Triple TOF 5600+, which is equipped with a DuoSpray ion source, was applied to detect products with high resolution (AB SCIEX, Foster City, CA, USA).

Fabrication of the CuO@GF electrode. CuO was prepared by calcination of $Cu(NO_3)_2$ under 450 °C for 2 h. The purchased commercial graphite felts (GF) with a thickness of 2.0 mm were washed by acetone, ethanol and DI water, sequentially. After drying at 60 °C for 6 h, the cleaned GF was tailored into the required structure for further use. 200 mg of prepared CuO was mixed

well with 20 mL ethanol, 20 mL DI water, and 200 μL Nafion solution (5 wt%). The mixture was ultrasonicated for 1.0 hour to obtain a stable suspension. After that, the suspension was evenly dropping-coated onto the prepared GF substrate with size of 3.0 cm× 2.0 cm × 0.2 cm (Length × Height × Thickness). The obtained sample was dried at 40 ºC for 6 h, before washing with DI water for several times. After that, the cleaned sample was used directly for further experiment.

Fabrication of the CNTs@GF electrode. In a typical experiment, the purchased commercial graphite felts (GF) with a thickness of 2.0 mm were washed by acetone, ethanol and DI water, sequentially. After drying at 60 °C for 6 h, the cleaned GF was tailored into the required structure for further use. 200 mg of CNTs was mixed well with 20 mL ethanol, 20 mL DI water, and 200 μL Nafion solution (5 wt%). The mixture was ultrasonicated for 1 h to obtain a stable suspension. After that, the suspension was evenly dropping-coated onto the prepared GF substrate with size of 3.0 cm× 2.0 cm × 0.20 cm (Length × Height × Thickness). The obtained sample was dried at 40 ºC for 6 h, before washing with DI water for several times. After that, the cleaned sample was used directly for further experiment.

BPA degradation under Cu-N-C powder catalyst. In a typical experiment, 25 mL of 0.1 mM BPA simulated wastewater was added into a 50 mL glass bottle, 0.15g L^{-1} Cu-N-C and 1 mM PDS were added under magnetic stirring. At regular time intervals, 1 mL of the mixture solution was fetched from the reactor and then filtered with a 0.22 µm PTFE filter. The collected solution was sent for HPLC analysis. The detailed detection parameters were presented in the Supplementary Table 1. Experiments were conducted in triplicate, and the error bars represent the arithmetic mean ± standard deviation.

Double-chamber reaction with CuO@GF electrode. The double-chamber reaction was conducted using a double-chamber reactor with interconnection section sealed with the proton exchange membrane. In a typical degradation experiment, 40 mL of 5 μM BPA solution and 40 mL of 10 mM PDS solution were added respectively into the anodic (right) and cathodic (left) chambers. Two tailored CuO@GF electrodes (3.0 cm× 2.0 cm × 0.2 cm, Length × Height × Thickness) were immersed into the solutions, respectively, with top ends connected by a copper wire. At scheduled reaction time intervals, 1 mL solution of the solution in anodic chamber was fetched and filtered with 0.22 μM PTEE membrane before testing with high-performance liquid chromatography (HPLC). Experiments were conducted in triplicate, and the error bars represent the arithmetic mean ± standard deviation.

Double-chamber reaction with CNTs@GF electrode. The double-chamber reaction was conducted using a double-chamber reactor with interconnection section sealed with the proton exchange membrane. In a typical degradation experiment, 40 mL of 5 μM BPA solution and 40 mL of 10 mM PDS solution were added respectively into the anodic (right) and cathodic (left) chambers. Two tailored CNTs@GF electrodes (3.0 cm× 2.0 cm × 0.20 cm, Length × Height ×

Thickness) were immersed into the solutions, respectively, with top ends connected by a copper wire. At scheduled reaction time intervals, 1 mL solution of the solution in anodic chamber was fetched and filtered with 0.22 μM PTEE membrane before testing with high-performance liquid chromatography (HPLC). Experiments were conducted in triplicate, and the error bars represent the arithmetic mean ± standard deviation.

Electrochemical analysis of CuO@GF and CNTs@GF based system. The electrochemical measurements were conducted via a computer-controlled potentiostat (CHI660E, C17171). Silver/silver chloride electrode (Ag/AgCl) and a carbon rod electrode were used as a reference electrode and a counter electrode, respectively. CuO@GF or CNTs@GF was served as a working electrode in a typical measurement. Electrolyte was 40.0 mM boric buffer with pH of 7.4. The open circuit potential was monitored by chronopotentiometry analysis with the addition of 1 mM PDS or 0.1 mM pollutants. The corresponding currents were recorded when an electrical potential of CuO@GF was set at +0.33 or +0.63 V or an electrical potential of CNTs@GF was set at +0.37 or +0.6 V, using the chronoamperometry (constant potential) measurement with/without 0.1 mM BPA addition in the electrolyte.

Electrochemical analysis of the powder catalyst-based system. Cu-N-C (2 mg) was first dispersed in 20 μL of nafion perfluorinated resin solution (5 wt %, Aldrich) and 2 mL ethanol, then ultrasonic dispersion for 30 min to form a suspension solution. The mixture (5 μL) was dropped onto a glassy carbon electrode and dried at 40 °C, and this procedure was repeated three times to prepare a working electrode. Silver/silver chloride electrode (Ag/AgCl) and Pt wire electrode were used as reference electrode and counter electrode, respectively. Chronoamperometries were carried out with the working electrode biased at an applied potential of +0.01 V (vs. Ag/AgCl) and electrochemical measurements were subsequently added into reactor at stated intervals with final concentrations of 1 mM PDS and 0.1 mM BPA, respectively. The open circuit potential was monitored by chronopotentiometry analysis with the addition of 1 mM PDS or 0.1 mM pollutants.

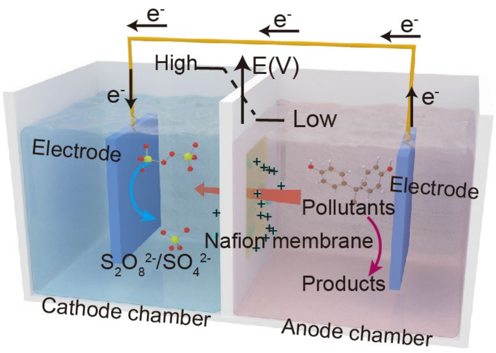

Supplementary Figure 1 | Scheme for a decoupled oxidation process (DOP) conducted in a double-chamber cell.

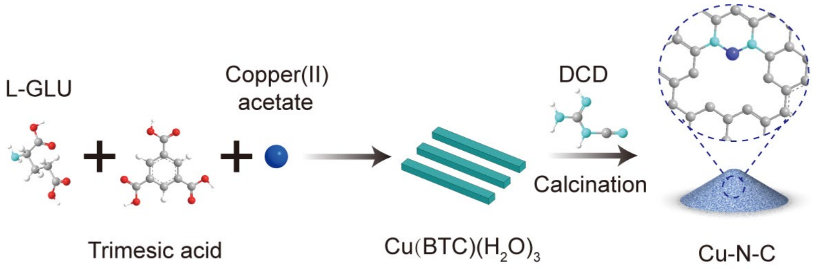

Supplementary Figure 2 | Schematic illustration for the synthesis process of Cu-N-C. L-GLU refers to L-glutamic acid and DCD refers to dicyandiamide.

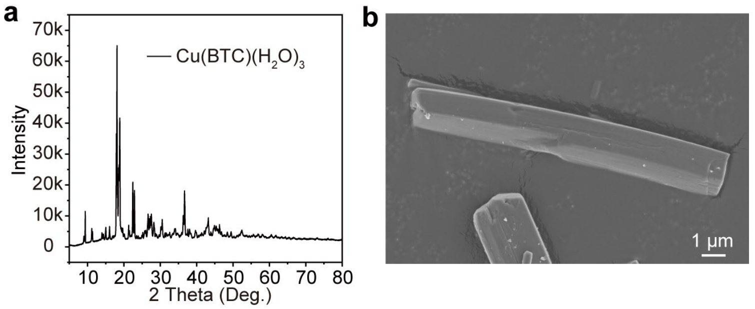

Supplementary Figure 3 | Characterization of $Cu(BTC)(H_2O)_3$. **a**, XRD pattern of $Cu(BTC)(H_2O)_3$. **b**, SEM images of the obtained $Cu(BTC)(H_2O)_3$.

The X-ray diffraction (XRD) pattern in Supplementary Figure 3a matches well with the structure of $Cu(BTC)(H_2O)_3$, unveiling the successful fabrication of $Cu(BTC)(H_2O)_3$[1,2]. The corresponding SEM images in Supplementary Figure 3b showed the rod-like structure of the products, which also matches well with the rod-like structure of $Cu(BTC)(H_2O)_3$.

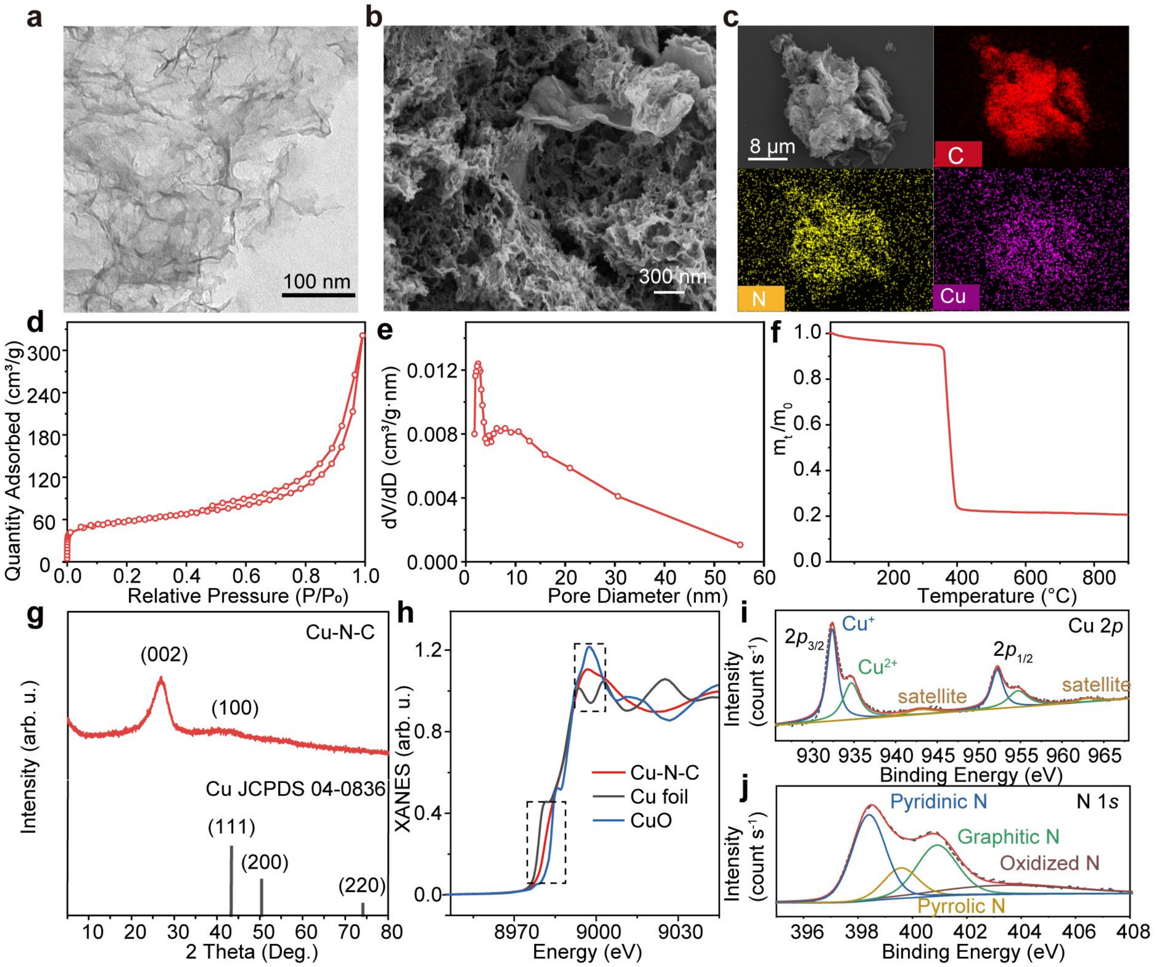

Supplementary Figure 4 | Characterization of Cu-N-C. a, TEM image of Cu-N-C. **b**, SEM image of prepared Cu-N-C. **c**, Energy dispersive X-ray spectroscopy (EDS) mappings of Cu-N-C. **d**, The N_2 adsorption-desorption isotherms of Cu-N-C. **e**, The pore size distributions of Cu-N-C. **f**, TGA result of Cu-N-C. **g,** XRD pattern spectrum of Cu-N-C. **h**, Normalized XANES of the samples at the Cu K-edge. **i**, The XPS pattern of Cu 2*p* of Cu-N-C. **j**, The XPS pattern of N 1*s* of Cu-N-C.

The low-resolution transmission electron microscopy (LRTEM) result in Supplementary Figure 4a indicated the two-dimensional ultrathin nanosheet structure of the obtained Cu-N-C, which didn't show presence of any crystallized particles, suggesting the possible atomically dispersed Cu sites. The SEM images of Cu-N-C (Supplementary Figure 4b) unveiled its porous structure, while the electron mapping via energy dispersive X-ray spectroscopy (EDS) (Supplementary Figure 4c) confirmed the uniform dispersion of N, C and Cu elements within the Cu-N-C composite.

The specific surface area of Cu-N-C was measured by nitrogen adsorption/desorption using the Brunauer-Emmett-Teller (BET) method (Supplementary Figure 4d, e). As measured, the Cu-N-C exhibited a specific surface area of 200 m^2 g^{-1}, with a total pore volume of 0.4926 cm^3 g^{-1}. Loading amount of Cu in the sample was analyzed via thermogravimetric analysis (TGA), which indicating an approximately 16.37 wt% loading amount of atomically dispersed Cu (Supplementary Figure 4f), suggesting the high loading density of Cu atoms on the composite.

The X-ray diffraction (XRD) measurement result (Supplementary Figure 4g) only presents the peak related to the graphitic structure of the carbon matrix, indicating the absence of formation of Cu based nanoparticles. The Cu K-edge X-ray absorption near-edge structure (XANES) spectrum in Supplementary Figure 4h showed the absorption edge and transition energies of Cu-N-C were located between Cu foil and CuO, indicating that the oxidation valence state of Cu atom was higher than 0 and lower than +2. The X-ray photoelectron spectroscopy (XPS) analysis (Supplementary Figure 4i, j) indicated that Cu existed as Cu^{+} and Cu^{2+} in the composite, and Cu-N-C contains a high concentration of pyridinic N site.

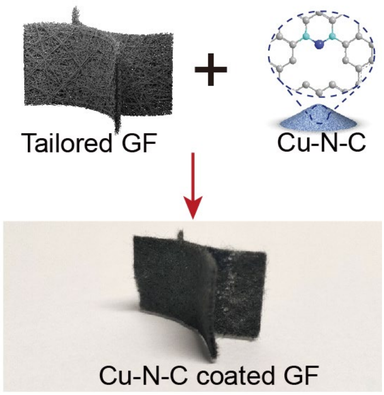

Supplementary Figure 5 |Schematic diagram for the preparation of Cu-N-C@GF electrode. The photograph at the bottom showed the prepared Cu-N-C@GF electrode. GF refers to graphitic felt.

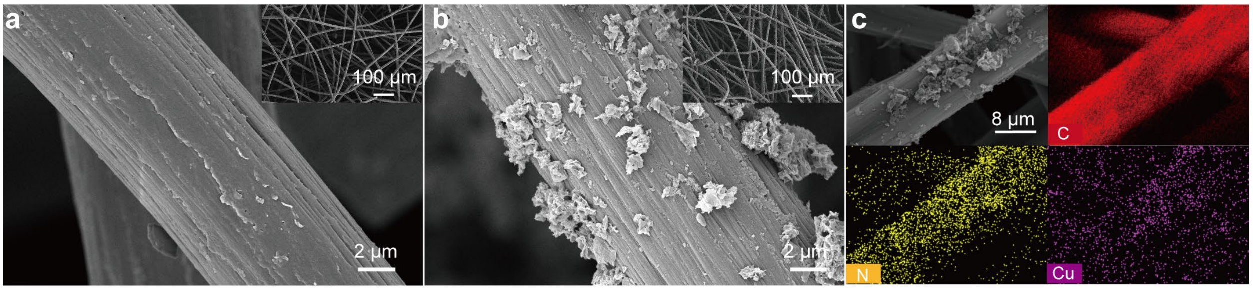

Supplementary Figure 6 | Characterization of Cu-N-C@GF. a, SEM images of GF. **b**, SEM images of Cu-N-C@GF. **c**, The C, N and Cu element mapping of Cu-N-C@GF.

The graphite felt before and after coating of Cu-N-C were analyzed via SEM. It can be seen that (Supplementary Figure 6a, b), the original graphite felt existed with a smooth surface, the presence of irregular particles on the fibers after coating indicated the successful loading of Cu-N-C. The insert SEM images in Supplementary Figure 6a and b indicated that the loading didn't affect the morphology of original carbon felt. The corresponding EDS mapping pattern of Cu-N-C@GF further confirmed the successful loading of Cu-N-C on graphite felt.

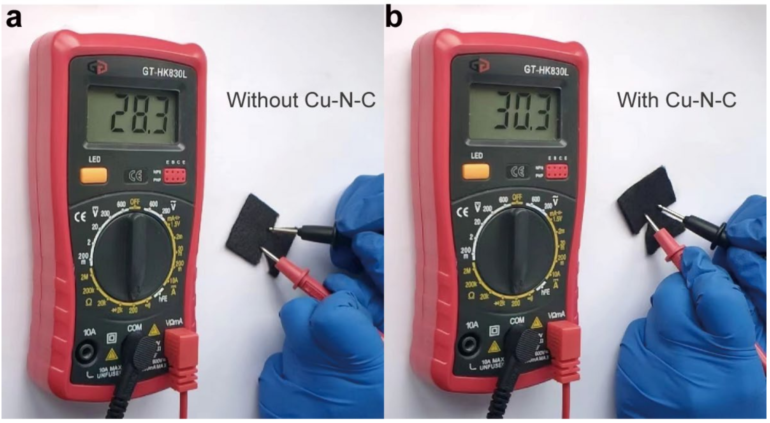

Supplementary Figure 7 | Resistance comparison of the samples. a, Resistance measurement of GF and **b**, Resistance measurement of Cu-N-C@GF.

The resistance of the graphite felt before and after loading the catalyst Cu-N-C was measured in Supplementary Figure 7, which indicating that loading of Cu-N-C didn't affect the electrode resistance obviously.

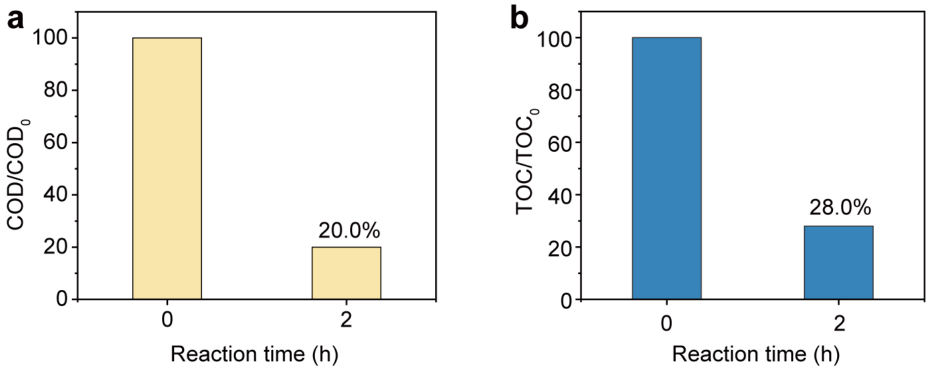

Supplementary Figure 8 | COD and TOC measurement. a, Comparison of the COD values before and after a 2-hour degradation of BPA. **b**, Comparison of the TOC values before and after a 2-hour degradation of BPA. Conditions: catalyst: 0.15 g L^{-1}, BPA: 0.1 mM, PDS: 1 mM.

As can be seen, both the COD and TOC values of the solution were efficiently decreased to approximately 20%, in comparison with the untreated solution, suggesting the good organic mineralization capability of the catalytic system.

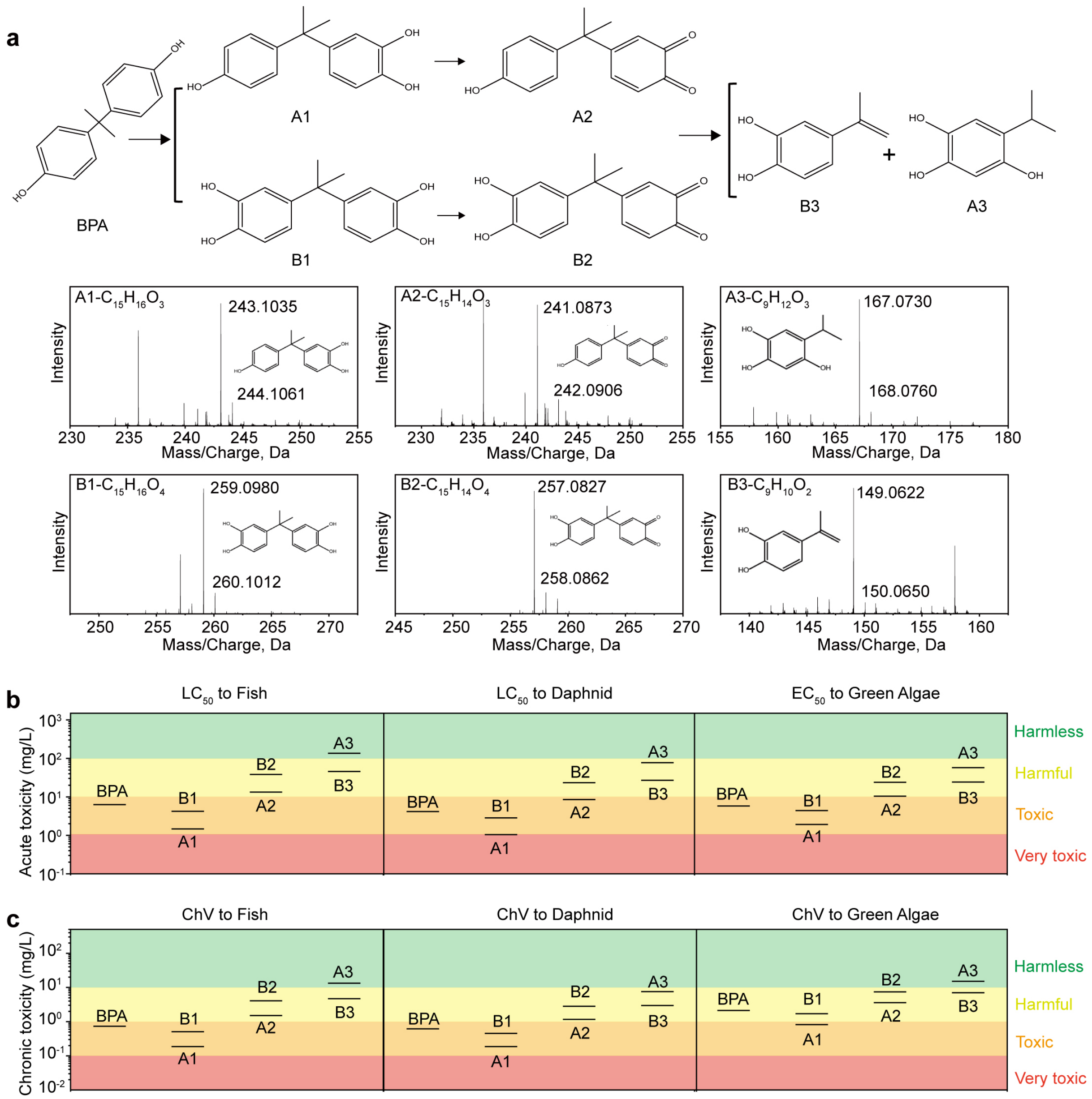

Supplementary Figure 9 | Products analysis and their corresponding toxicity assessment. a, Proposed degradation pathway of BPA based on the detected intermediate products. **b** and **c**, The respective acute and chronic toxicity analysis of BPA and its corresponding degradation products with the ecological structure-activity relationship model (ECOSAR). Green, yellow, orange and red represent harmless, harmful, means toxic and highly toxic respectively. LC_{50}: lethal concentration 50; EC_{50}: concentration for 50% of maximal effect; ChV: chronic value.

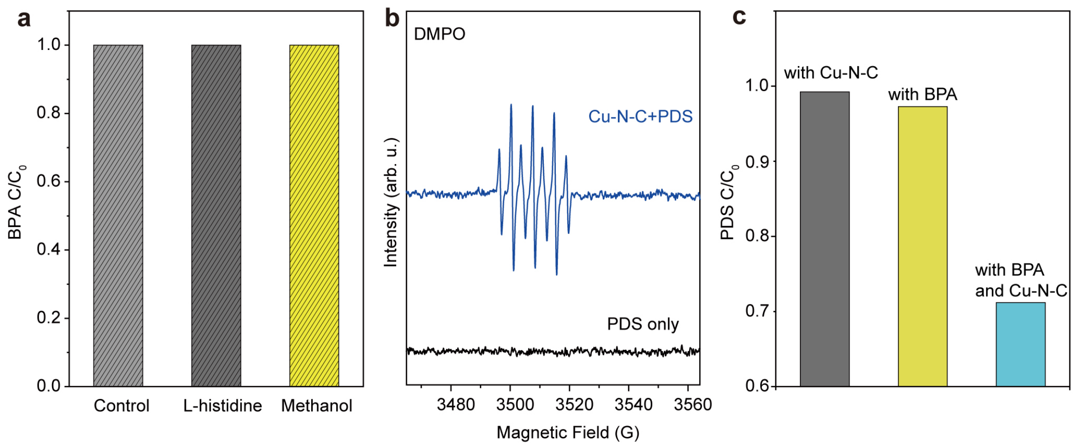

Supplementary Figure 10 | Investigation of reaction mechanism. **a**, Comparison of reaction rate with different scavengers. **b**, EPR spectra by using DMPO as the trapping agent. **c**, Decomposition of PDS in the presence of Cu-N-C and BPA. Reaction condition (if required): BPA: 0.1 mM; PDS: 1 mM; Cu-N-C: 0.15 g L^{-1}; L-histidine: 5 mM; methanol: 100 mM; DMPO: 50 mM.

As shown in Supplementary Figure 10a, the addition of methanol (MeOH) (scavenger of $SO_4^{\bullet-}$, •OH) and L-histidine (scavenger of $^1O_2$) didn't cause obvious change on the BPA removal efficiency, which suggests that the active radicals $SO_4^{\bullet-}$, •OH and $^1O_2$ didn't exist in our system. Additionally, 5,5-dimethyl-pyridine N-oxide (DMPO) was used as the spin-trapping reagent to further verify this (Supplementary Figure 10b). Unlike the signals of DMPO-•OH or DMPO-$SO_4^{\bullet-}$ adducts, a characteristic signal of 5,5-dimethyl-2-pyrrolidone-N-oxyl (DMPOX) was detected after adding Cu-N-C, unveiling that active radicals are not existed, but an oxidation reaction occurs. In Supplementary Figure 10c, we further tested the concentration change of PDS under different situation. As can be seen, there was no obvious change in PDS concentration with the respective existence of Cu-N-C and PDS in the system, while the PDS concentration decreased obviously with the co-existence of Cu-N-C and BPA.

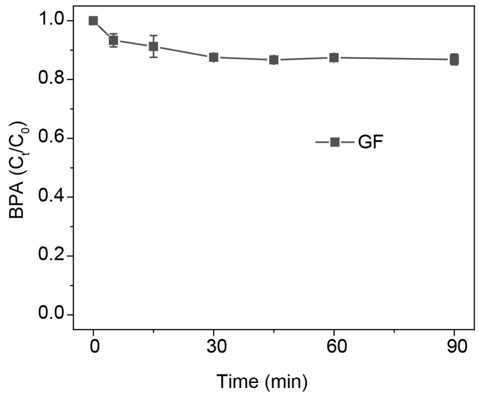

Supplementary Figure 11 | Performance evolution of bare graphitic felt. BPA degradation in the double-chamber reactor with the pure graphite felt. Reaction condition: BPA: 5 μM; NaCl: 0.5 wt‰. Experiments were conducted in triplicate, and the error bars represent the arithmetic mean ± standard deviation.

Supplementary Figure 11 showed the removal of BPA in the double-chamber reactor when the pure graphite felt was as the electrode, suggesting BPA couldn't be removed in 90 mins without Cu-N-C.

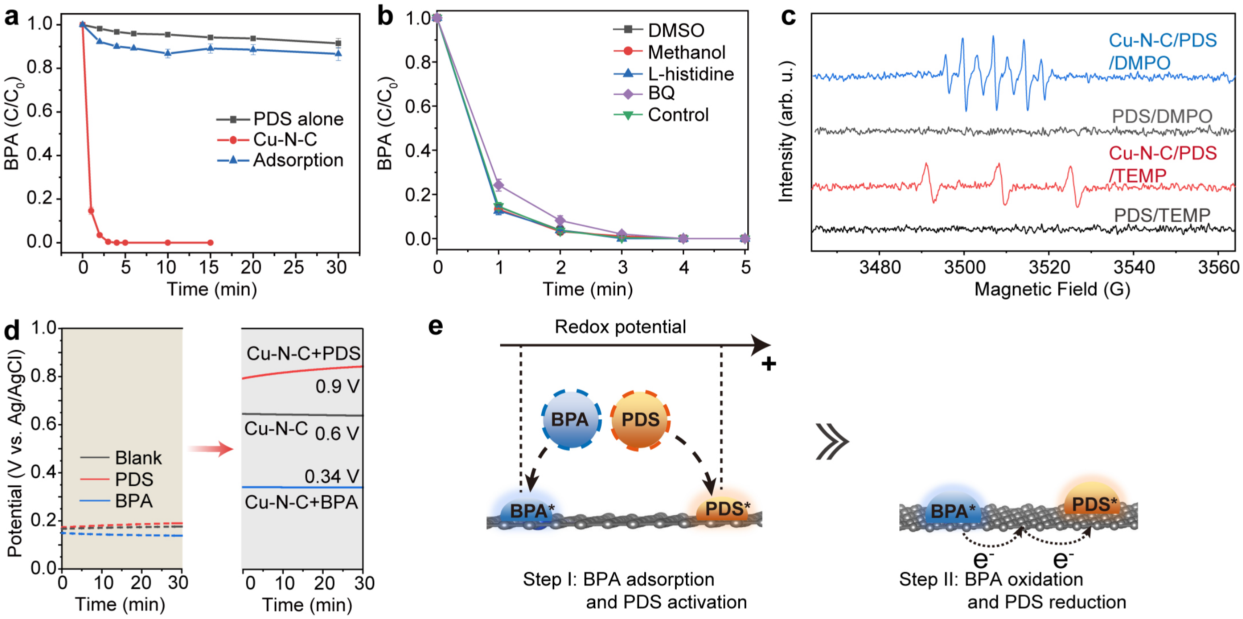

Supplementary Figure 12 | Investigation of reaction mechanism involved in the powder catalyst Cu-N-C mixed solution. a, The monitored BPA concentration change under different condition. Conditions (if required): catalyst: 0.15 g L^{-1}, BPA: 0.1 mM, PDS: 1 mM. **b**, Monitored BPA removal performance under the presence of difference scavengers. Conditions (if required): catalyst: 0.15 g L^{-1}, BPA: 0.1 mM, PDS: 1 mM, L-histidine: 50 mM, Methanol (MeOH): 500 mM, Dimethyl sulfoxide (DMSO) = Benzoquinone (BQ): 5 mM. **c**, The measured EPR spectra with DMPO and TEMP as the trapping agent, respectively. Conditions (if required): catalyst: 0.15 g L^{-1}, BPA: 0.1 mM, PDS: 1 mM, DMPO = TEMP: 50 mM. **d**, The Open-circuit potential curve measurement of BPA (0.1 mM) and PDS (1 mM) solutions with different triple electrode system. Reference electrode: silver/silver chloride electrode (Ag/AgCl). Counter electrode: platinum electrode. Working electrodes: glassy carbon electrode coated with/without Cu-N-C. **e**, Scheme illustration of the electron transfer process involved in the oxidation of BPA under the assistance of powder Cu-N-C catalyst and PDS. Experiments in **a** and **b** were conducted in triplicate, and the error bars represent the arithmetic mean ± standard deviation.

Supplementary Figure 12a showed that the catalytic activation of PDS played the main role for BPA removal. As presented in Supplementary Figure 12b, the addition of quenching agents showed little influence on BPA removal, suggesting that the oxidation of BPA in the PDS/Cu-N-C system did not rely on the radical species $SO_4^{\bullet -}$, $^{\bullet}OH$, $O_2^{\bullet -}$ and $^1O_2$[3,4]. A further EPR analysis results presented on Supplementary Figure 12c also confirmed the absence of the above radicals in the reaction.

Supplementary Figure 12d revealed that the presence of Cu-N-C created a significant potential difference (ΔE = 0.9-0.34 = 0.56 V) between the absorbed PDS* and BPA*, driving electron transfer and resulting in BPA oxidation, as depicted in the schematic diagram in Supplementary Figure 12e.

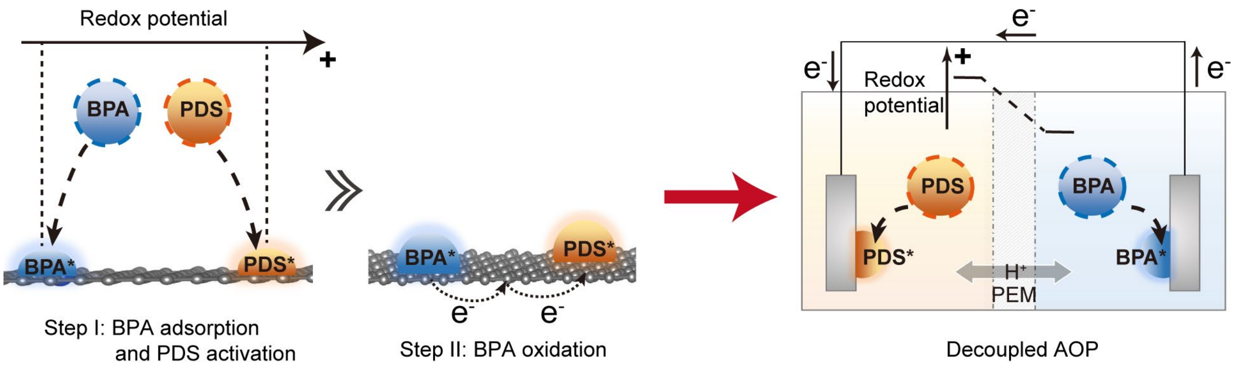

Supplementary Figure 13 | Schematic diagram of the concept design derived from the powder catalyst to the DOP set.

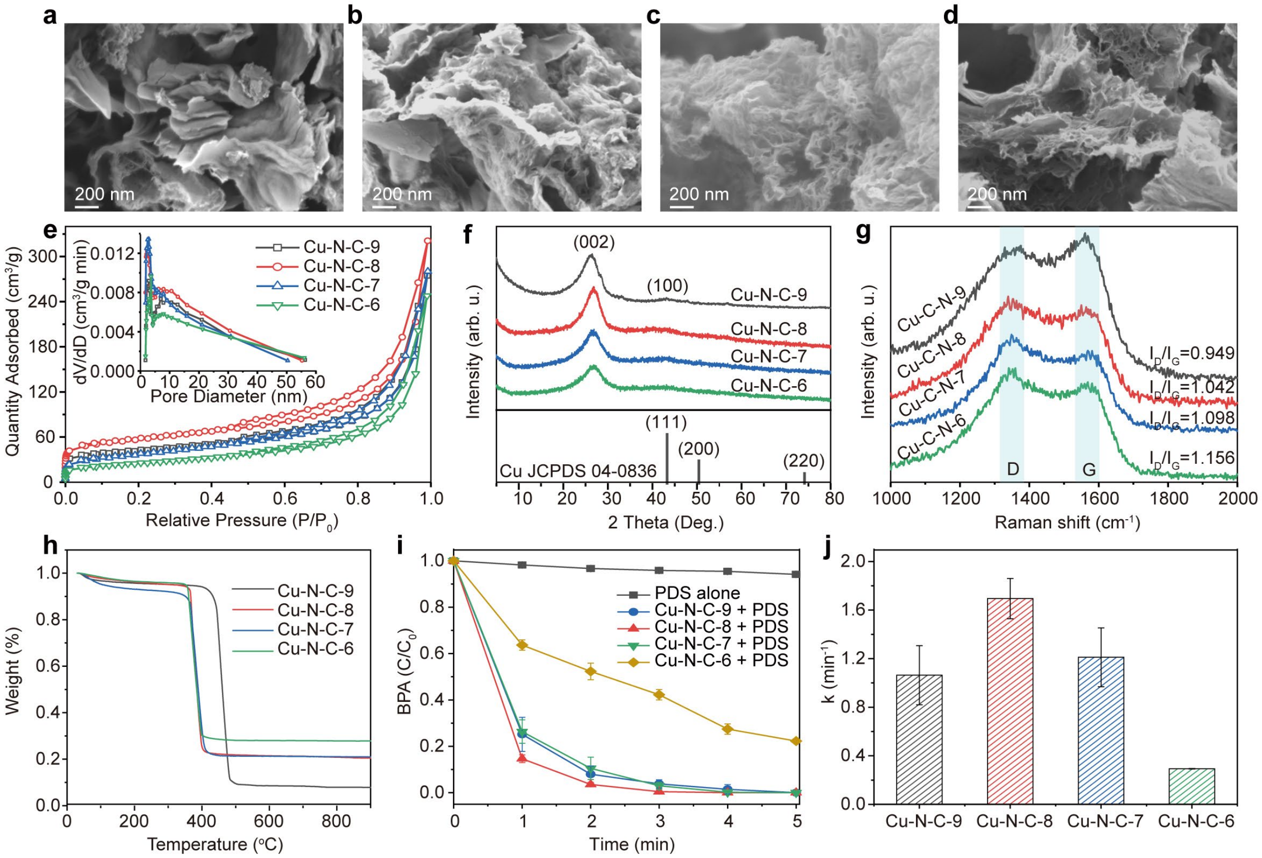

Supplementary Figure 14 | Characterization and performance analysis of the Cu-N-C-n series. n (=6, 7, 8 and 9) refers to the sample prepared under 600, 700, 800 and 900 ºC calcination temperature. **a-d**, The respective low-resolution SEM images of Cu-N-C-n. **e**, The measured N_2 adsorption-desorption isotherms and pore size distributions (the inset graph) of Cu-N-C-n. **f**, The measured XRD spectra of Cu-N-C-n. **g**, The measured Raman spectra of Cu-N-C-n. **h**, The measured TGA results of Cu-N-C-n. The Cu element of catalysts can be calculated by multiplying the rest of mass ratio and the Cu amount of CuO (product in high temperature, 79.9 wt%). **i**, The monitored BPA concentration change in different systems. Conditions (if required): catalyst: 0.15 g L^{-1}, BPA: 0.1 mM, PDS: 1.0 mM. **j**, The calculated k values in correspondence of i. Experiments in **i** and **j** were conducted in triplicate, and the error bars represent the arithmetic mean ± standard deviation.

The precursor ($Cu(BTC)(H_2O)_3$ MOF and DCD) was calcined at 600 ºC, 700 ºC, and 900 ºC, resulting in composites named Cu-N-C-6, Cu-N-C-7, and Cu-N-C-9, respectively. The catalyst used to demonstrate the design concept was calcined at 800 ºC and referred to as Cu-N-C-8 for clarity.

The four composites were adopted to catalyze the oxidation of BPA with mixing with PDS and BPA respectively in an all-in-one solution-based reaction system (Supplementary Figure 14i and j). Cu-N-C-8 exhibited exceptional BPA removal performance, reaching close to 100% within 4 minutes, with a k value around 1.78 min^{-1}, surpassing most reported literature (Supplementary Table 4). Via the above analysis, we can attribute the excellent catalytic performance of Cu-N-C-8 to its porous structure with relatively large surface area (~199.59 m^2 g^{-1}), as well as the highest atomically dispersed Cu atoms (Supplementary Figure 14h). Therefore, we can conclude that the atomically dispersed Cu sites may mainly serve as the active sites for reaction. Since composite Cu-N-C-8 showed the best catalytic performance, it was selected as the catalyst for further experiment and we simplified its name to Cu-N-C in the following sections.

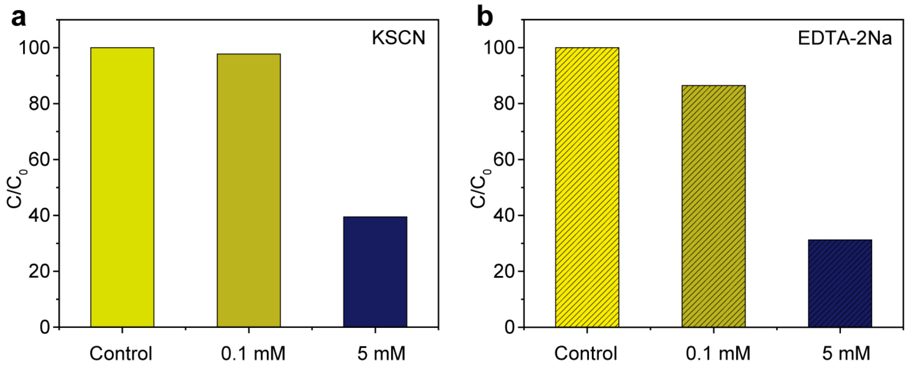

Supplementary Figure 15 | Investigation of role of Cu atoms for the catalytic reaction. a, Complexation of catalyst by KSCN. **b**, Complexation of catalyst by EDTA-2Na. Reaction condition (if required): BPA: 0.1 mM, PDS: 1 mM, Cu-N-C: 0.15 g L^{-1}.

KSCN and EDTA-2Na, as two representative chelating agents for Cu ions[5], were added into the Cu-N-C/PDS/BPA system to evaluate the role of Cu-N species, respectively. Obviously, 0.1 mM chelating agents reduced BPA degradation rates and greatly suppressed BPA degradation when 5 mM KSCN and EDTA-2Na were independently added, indicating the Cu-N sites were mainly responsible for the catalytic reaction.

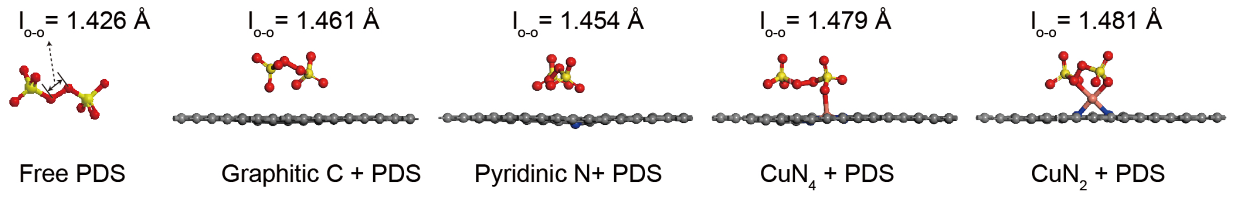

Supplementary Figure 16. Simulation of contact process between PDS and reaction sites. Orange, Cu; blue, N; white, H; grey, C; red, O; yellow, S.

As shown in Supplementary Figure 16, the O-O bond stretching (l_{o-o}) of PDS was weakened and significantly lengthened to 1.479 Å and 1.481 Å on the CuN_4 and CuN_2, respectively, much higher than that of the free PMS molecule, graphitic C and pyridinic N. The results indicated that CuN_2 and CuN_4 on Cu-N-C were the ideal sites for activation of PDS.

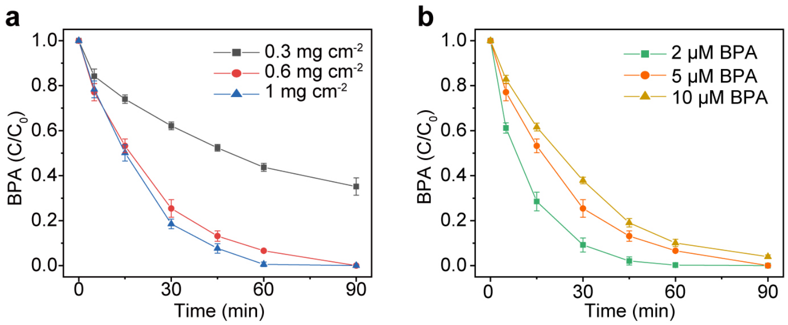

Supplementary Figure 17 | Performance investigation under different reaction condition. a, The measured BPA removal performance in the DOP two-chamber reactor, with different catalyst loading amount on the graphite felt electrode. Conditions: BPA: 5 μM, PDS: 10 mM, NaCl: 0.5 wt‰. **b**, The measured BPA removal performance in the DOP two-chamber reactor, towards different BPA concentration. Conditions: PDS: 10 mM, NaCl: 0.5 wt‰. Experiments in **a** and **b** were conducted in triplicate, and the error bars represent the arithmetic mean ± standard deviation.

The loading amount of catalyst Cu-N-C on graphite felt electrode was tuned (shown as Supplementary Figure 17a). As can be seen, a first increase of the loading catalyst amount from 0.3 to 0.6 mg cm^{-2} resulted in significant boosted BPA removal efficiency, however, a further increased loading amount to 1 mg cm^{-2} didn't contribute to obvious improvement. Therefore, the catalyst loading amount 0.6 mg cm^{-2} on the electrode was adopted for the further experiment. We further test the removal efficiency of our reactor towards BPA solution in different concentration. As shown in Supplementary Figure 17b, our DOP presented a rapid removal of BPA, in a wide concentration range from 2 μM to 10 μM, which might suggest the application of this set for the treatment towards different concentrations of pollutants.

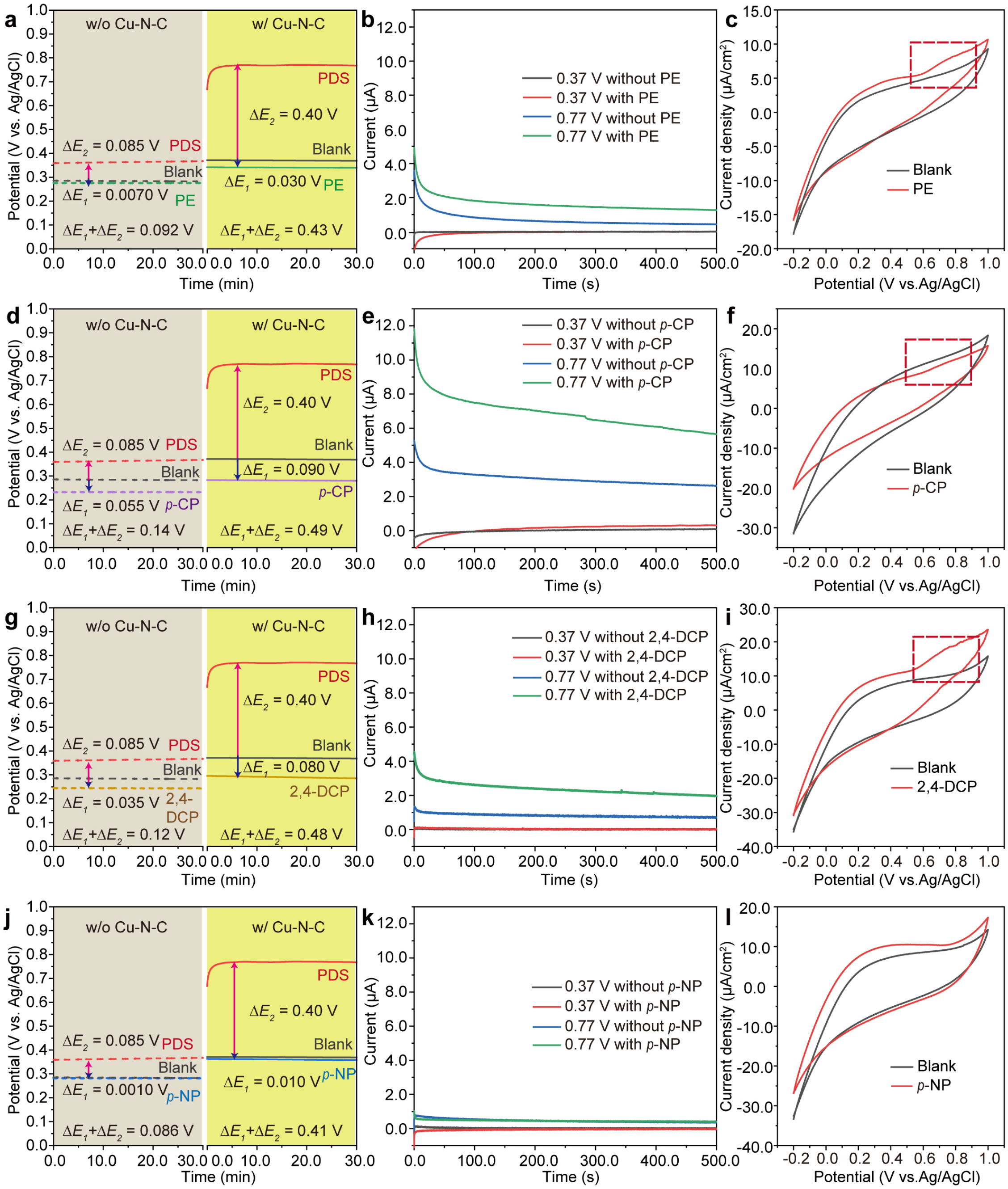

Supplementary Figure 18. Electrochemical tests for different pollutant systems. Reaction condition: PDS: 10 mM; *p*-NP: 5.0 μM; phenol: 5 μM; *p*-CP: 5 μM; 2,4-DCP, 5 μM; NaCl: 0.5 wt‰.

Supplementary Figure 18 shows the electrochemical characterization of different pollutants. For phenol (PE), *p*-chlorophenol (*p*-CP) and 2, 4-dichlorophenol (2,4-DCP), the addition of pollutants significantly decreased the potential of Cu-N-C@GF (Supplementary Figure 18a, d and

g), while the addition of *p*-nitrophenol (*p*-NP) couldn't significantly change the potential of Cu-N-C@GF (Supplementary Figure 18j). At the applied voltage of +0.37 V (the initial potential of the Cu-N-C@GF), there was little difference in the current with or without pollutants. However, when the given potential reached the equilibrium potential of PDS/ Cu-N-C@GF (+0.77 V), the current intensity of systems containing PE, *p*-CP and 2,4-DCP was significantly higher than that of systems without them (Supplementary Figure 18b, e and h), whereas there was still no change for *p*-NP (Supplementary Figure 18k). It was suggested that the voltage of the Cu-N-C@GF alone without PDS was not enough to oxidize pollutants, and a PDS/Cu-N-C@GF complex formed from activating PDS would elevate the potential of Cu-N-C@GF, which could oxidize pollutants except *p*-NP. Similarly, PE, *p*-CP and 2,4-DCP had the oxidation peak in the cyclic voltammetry curve (Supplementary Figure 18c, f and i), different from *p*-NP (Supplementary Figure 18l), which matched well with the above results.

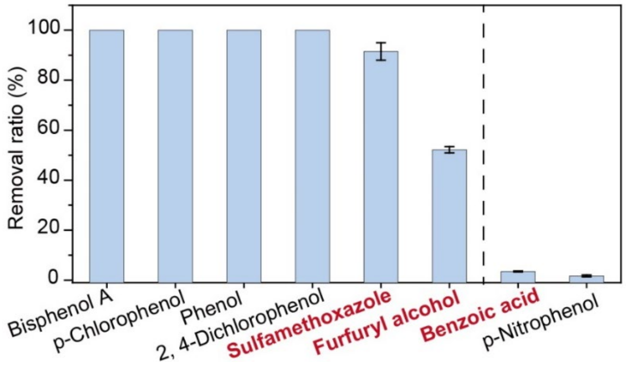

Supplementary Figure 19. Monitored concentration change of different organic pollutants using the double-chamber reactor. Conditions: pollutants: 5.0 μM. Experiments were conducted in triplicate, and the error bars represent the arithmetic mean ± standard deviation.

We investigated the potential of our strategy towards other types of organic pollutants (Supplementary Figure 19). As can be seen, our set only not show excellent removal efficiency towards phenolic compounds, but also to antibiotic compound sulfamethoxazole and heterocyclic compound furfuryl alcohol. The removal performance towards organic compound benzoic acid and p-nitrophenol is not good, we attribute this to the electro-withdrawing functional groups on the compounds to prevent the reactions[6-8]. The good removal efficiency of our system towards these organic compounds suggests its applicability for treatment of electro-rich organic compound impaired water.

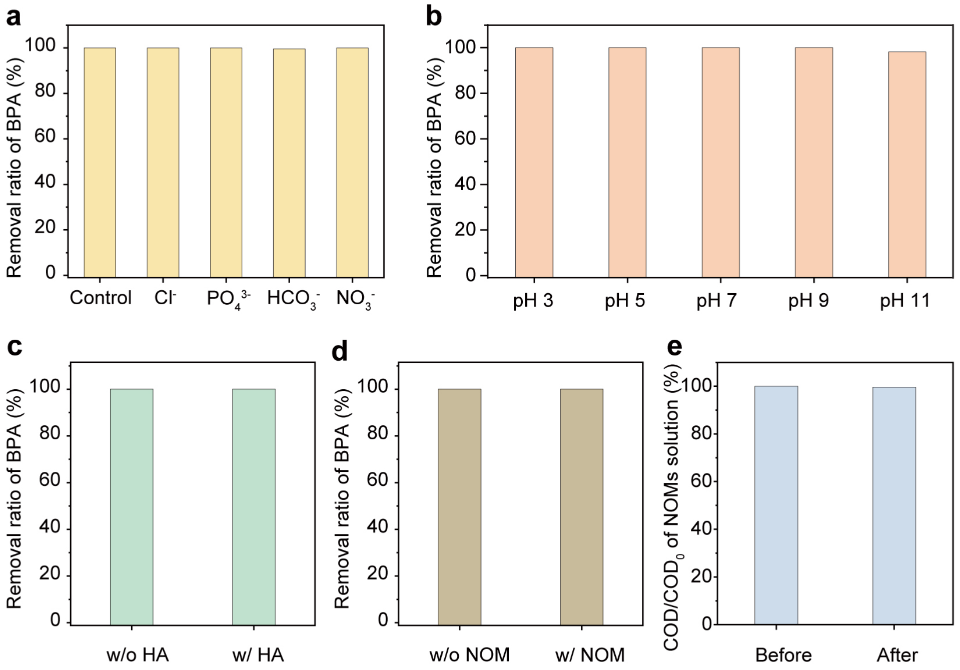

Supplementary Figure 20 | The influence of the natural factors on the reaction performance. **a**, The removal performance of BPA under the presence of different inorganic ions with the DOP system. Conditions: BPA: 5 μM, inorganic ions: 20 mM. **b**, The removal performance of BPA under different pH values. Conditions: BPA: 5 μM. **c**, Comparison of the removal performance of BPA with and without the presence of humic acid. Conditions: BPA: 5 μM, humid acid (HA): 5 ppm. **d**, Comparison of the removal performance of BPA with and without the addition of commercial simulated natural organic matters (NOMs, Upper Mississippi River NOM No.1R110N). Conditions: BPA: 5 μM, NOMs: 5 ppm. **e**, The measured COD change of NOMs dissolved aqueous solution before and after conducting the Cu-N-C@GF based DOP set. Conditions: NOMs: 5 ppm. Note that all the measurements were conducted after the treatment with Cu-N-C@GF based DOP set if without further notice.

The influence of typical inorganic ions in natural water, different pH values (range from pH 3 to 11) as well as the presence of humic acid (HA) on the BPA removal performance were measured and the results were summarized in Supplementary Figure 20 a-c. As can be seen, regardless of these factors, the removal efficiency of BPA consistently remains around 100%, underscoring the robustness of our system. To explore the influence of natural organic matters (NOMs) on the reaction performance, we purchased commercial simulated NOMs (Upper Mississippi River NOM No.1R110N) and mixed it with the BPA solution to conduct the degradation experiment (Supplementary Figure 20d). It can be seen that, the system maintains a consistently high BPA removal efficiency close to 100‰, irrespective of the presence of the

simulated NOMs. Moreover, we extended our investigation to measure the chemical oxygen demand (COD) change of a prepared NOMs solution (5 ppm) during a 2-hour reaction employing our DOP system. Encouragingly, the COD value of the solution remained unchanged before and after the reaction, providing clear evidence that no reaction occurred between the NOMs and the catalytic electrode.

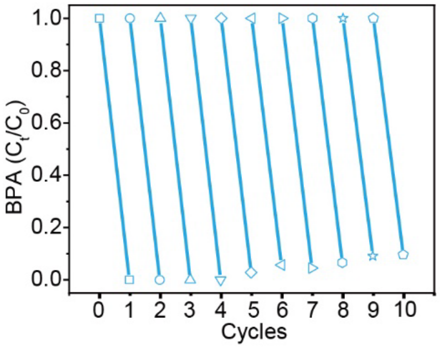

Supplementary Figure 21. Cycled degradation performance of our Cu-N-C@GF electrode for the BPA removal with the DOP reaction. Conditions: BPA: 5 μM, PDS: 10 mM, NaCl: 0.5 wt‰.

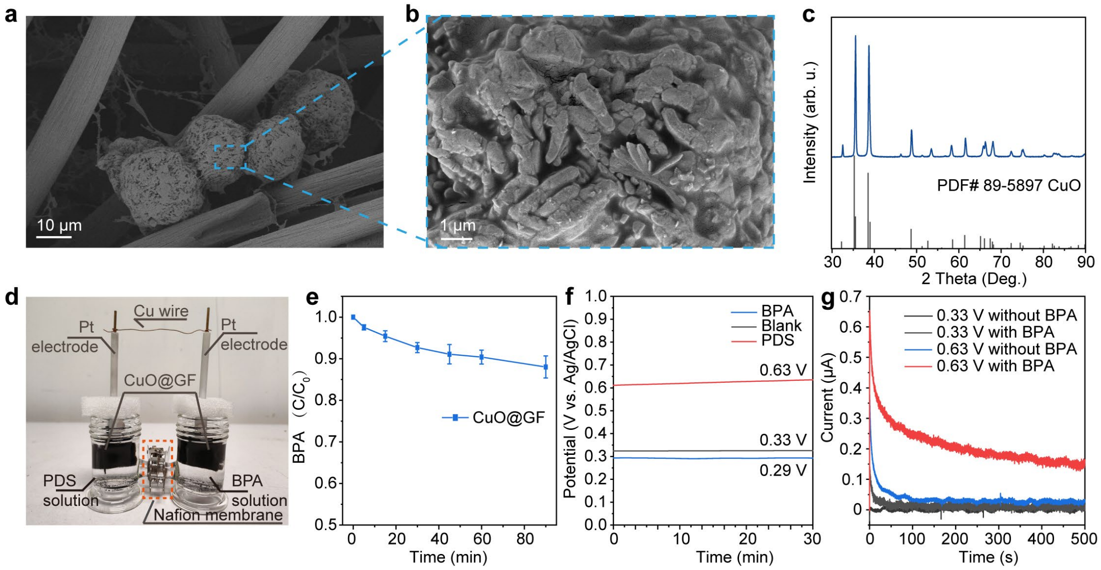

Supplementary Figure 22 | Characteristics and catalytic performance of CuO@GF. a, SEM image of CuO@GF. **b**, SEM image of CuO. **c**, The measured XRD pattern of CuO. **d**, Photograph of the double-chamber reactor we set to test performance of CuO@GF. **e**, The corresponding measured BPA removal performance with the set in d. Conditions: BPA: 5 μM, PDS: 10 mM, NaCl: 0.5 wt‰. Experiments were conducted in triplicate, and the error bars represent the arithmetic mean ± standard deviation. **f**, The measured open-circuit potential curve on the CuO@GF. Conditions: BPA: 0.1 mM, PDS: 1 mM. Reference electrode: silver/silver chloride electrode (Ag/AgCl). Counter electrode: a carbon rod electrode. Working electrodes: CuO@GF electrode. **g**, Measurement of current-time curves with different potentials on the CuO@GF electrode. Conditions: BPA: 0.1 mM, PDS: 1 mM. Reference electrode: silver/silver chloride electrode (Ag/AgCl). Counter electrode: a carbon rod electrode. Working electrodes: CuO@GF electrode.

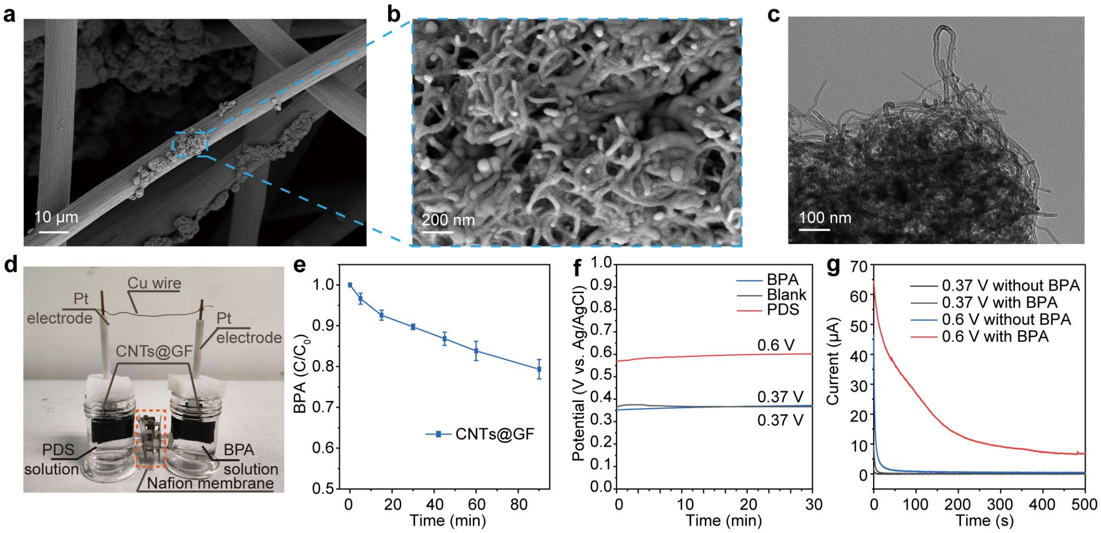

Supplementary Figure 23 | Characteristics and catalytic performance of CNTs@GF. a, SEM image of CNTs@GF. **b**, SEM image of CNTs. **c**, The measured XRD pattern of CNTs. **d**, Photograph of the double-chamber reactor we set to test performance of CNTs@GF. **e**, The corresponding measured BPA removal performance with the set in d. Conditions: BPA: 5 μM, PDS: 10 mM, NaCl: 0.5 wt‰. Experiments were conducted in triplicate, and the error bars represent the arithmetic mean ± standard deviation. **f**, The measured open-circuit potential curve on the CNTs@GF. Conditions: BPA: 0.1 mM, PDS: 1 mM. Reference electrode: silver/silver chloride electrode (Ag/AgCl). Counter electrode: a carbon rod electrode. Working electrodes: CNTs@GF electrode. **g**, Measurement of current-time curves with different potentials on the CNTs@GF electrode. Conditions: BPA: 0.1 mM, PDS: 1 mM. Reference electrode: silver/silver chloride electrode (Ag/AgCl). Counter electrode: a carbon rod electrode. Working electrodes: CNTs@GF electrode.

The CuO and CNTs were coated onto the graphite felt (GF) to prepare the CuO@GF and CNTs@GF electrodes, respectively (Supplementary Figure 22 and S23 above). The low-resolution SEM and XRD analysis were conducted respectively to show the successful coating of CuO and CNTs onto the electrode (Supplementary Figure 22a-c and Supplementary Figure 23a-c). By conducting the DOP reaction with the prepared CuO@GF and CNTs@GF electrodes, respectively (Supplementary Figure 22d and S23d), they both showed BPA removal capability in a certain degree (Supplementary Figure 22e and S23e). The respective chronopotentiometry analysis unveiled a ΔE_{CuO} around 0.34 V and ΔE_{CNTs} around 0.23 V existed in the two reaction systems (Supplementary Figure 22f and S23f). Furthermore, under the respectively applied voltages, the detected current further support the occurrence of the electrochemical catalytic reaction (Supplementary Figure 22g and S23g). The above experiment helped to demonstrate the generality of our DOP strategy.

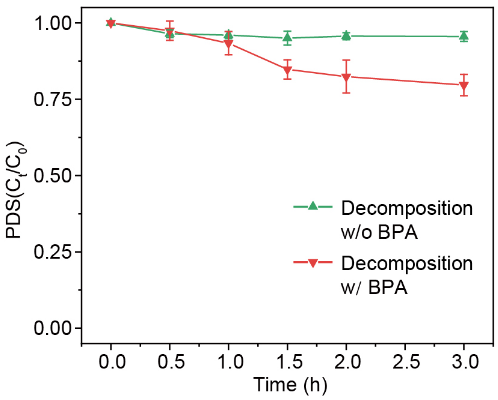

Supplementary Figure 24. Comparison of PDS concentration changes in the reactor under different conditions. Reaction condition (if required): PDS: 10 mM; BPA: 5 μM; NaCl: 0.5 wt‰. Experiments were conducted in triplicate, and the error bars represent the arithmetic mean ± standard deviation.

Supplementary Figure 24 illustrates the change of PDS concentration stored in the cylindrical tube during the experiment that the catalytic performance of the integrated reactor was measured when the electrodes was connected in experiment conducted in Figure 3c. With the continuous BPA removal reaction, PDS concentration decreased significantly. While in contrast, without the presence of BPA in the bottom water tank, the PDS concentration didn't show any significant change.

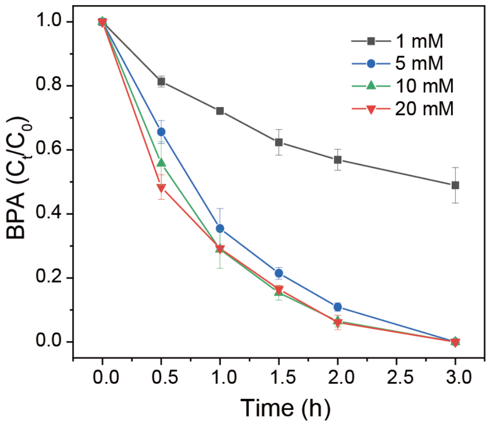

Supplementary Figure 25. The degradation curve of BPA at different concentrations of PDS. Reaction condition: BPA: 5.0 μM, NaCl: 0.5 wt‰. Experiments were conducted in triplicate, and the error bars represent the arithmetic mean ± standard deviation.

The effect of PDS concentration on the degradation of BPA was shown in Supplementary Figure 25, indicating the degradation rate of BPA in the tank decreased with the decrease of the initial concentration of PDS in the tube.

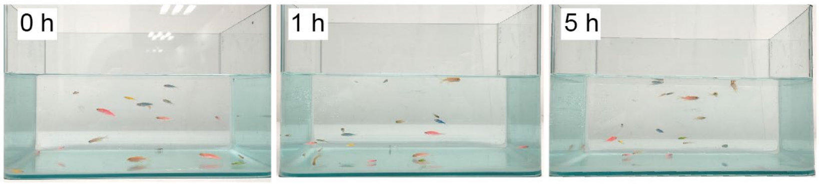

Supplementary Figure 26. Survival of fish in control group. Reaction condition: BPA: 5 μM; NaCl: 0.5 wt‰.

For the environment containing only 0.5 wt‰ NaCl, there was no obvious death of zebrafish as shown in Supplementary Figure 26, indicating that the initial environment would not cause the death of zebrafish within 5 hours.

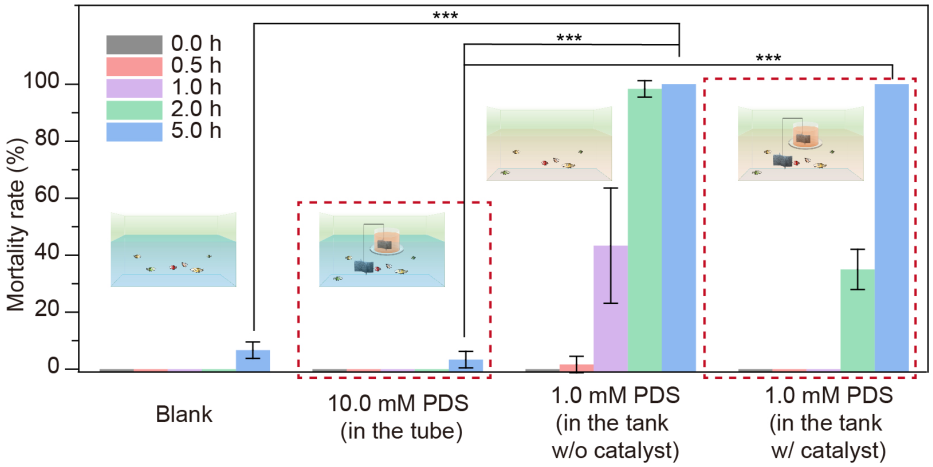

Supplementary Figure 27. The monitored mortality rate of zebrafish in different situation. For the four groups from left to the right, tank with BPA-contaminated wastewater, tank with BPA-contaminated wastewater equipped with a floating treatment device, tank with BPA-contaminated wastewater supplemented with PDS, and tank with BPA-contaminated wastewater supplemented with both PDS and a floating treatment device. Conditions (if required): BPA: 5 μM, PDS: 1 mM (in the tank)/10 mM (40 mL in the tube), NaCl:0.5 wt‰. The error bars in f were based on parallel experiments of three groups of zebrafish. NS refers to no signification. ***$p < 0.001$, **$p < 0.01$, or *$p < 0.05$. Experiments were conducted in triplicate, and the error bars represent the arithmetic mean ± standard deviation.

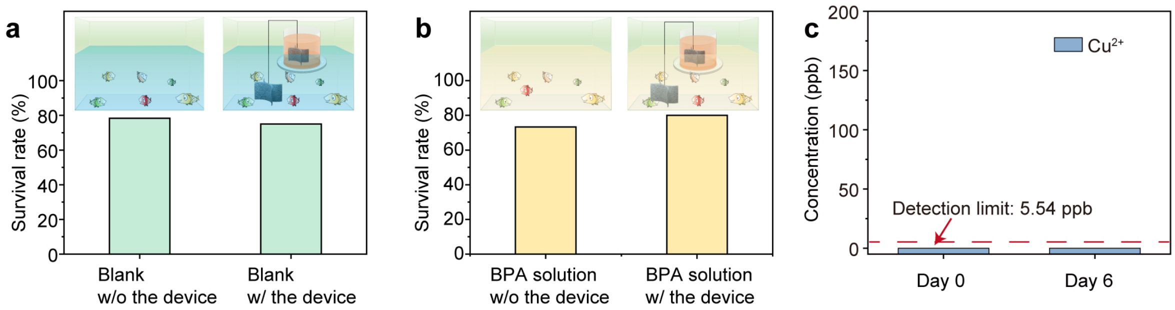

Supplementary Figure 28 | Long-term effects of the device on zebrafish. a, Survival of zebrafish with/ without the device for two weeks in the water contained 0.5 wt‰ NaCl. **b**, Survival of zebrafish with/ without the device for two weeks in the water contained 0.5 wt‰ NaCl and 5 μM BPA. Conditions: 10 mM PDS solution stored in the tube. **c**, The detected copper ion concentration in the tank in Supplementary Figure 28b before and after the 14-day continuous treatment.

In Supplementary Figure 28a, we assessed the environmental safety of our methodology within an aquatic context. Two separate tanks were utilized, each containing clean water and housing a population of 20 zebrafish. One of these tanks was equipped with our floating DOP device, while the other tank remained untreated as a control. Over a two-week observation period (Supplementary Figure 28a), we meticulously monitored zebrafish mortality rates. Notably, the presence of our AOP device did not result in any discernible increase in zebrafish mortality when compared to the control group.

Furthermore, we established two analogous tanks, however, we substituted the contents of the tanks with the BPA solution (5 μM), instead of clean water used previously (Supplementary Figure 28b). Notably, the tank containing the BPA solution with the integrated floating device exhibited a comparatively higher fish survival rate than the tank with the BPA solution lacking the treatment device. We attribute the diminished fish survival rate in the former tank to the toxic effects induced by BPA, whereas in the latter tank, the BPA concentration was progressively reduced by the device, resulting in fewer fish fatalities.

Additionally, we tested and compared the Cu^{2+} ion in the solution before and after the 14-day continuous experiment in Supplementary Figure 28b (Supplementary Figure 28c). The non-detectable Cu^{2+} ion concentration (both the values are far below the detection limit (5.54 ppb) of flame atomic absorption spectrometry) suggested the stability and safety of our electrode.

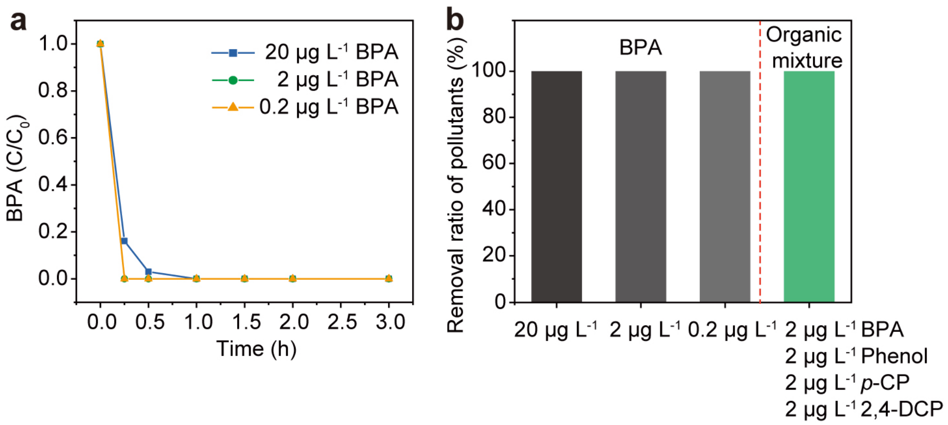

Supplementary Figure 29 | The catalytic performance of the device on different pollutant concentrations. a, Measured BPA removal performance with the floating device, towards 2 L water with different BPA concentrations. **b,** Calculated removal ratio of pollutants with the floating device after 3 h of reaction time. Note that the organic mixture was a 2 L solution of water containing 2 μg L^{-1} BPA, 2 μg L^{-1} phenol, 2 μg L^{-1} *p*-chlorophenol (*p*-CP) and 2 μg L^{-1} 2,4-dichlorophenol (2,4-DCP). Conditions: PDS = 10 mM, NaCl = 0.5 wt‰.

The consistent removal efficacy of the floating device across all three concentrations (20, 2, and 0.2 μg L^{-1}), as well as when challenged with a mixture of organic compounds including BPA, phenol, p-CP, and 2,4-DCP.

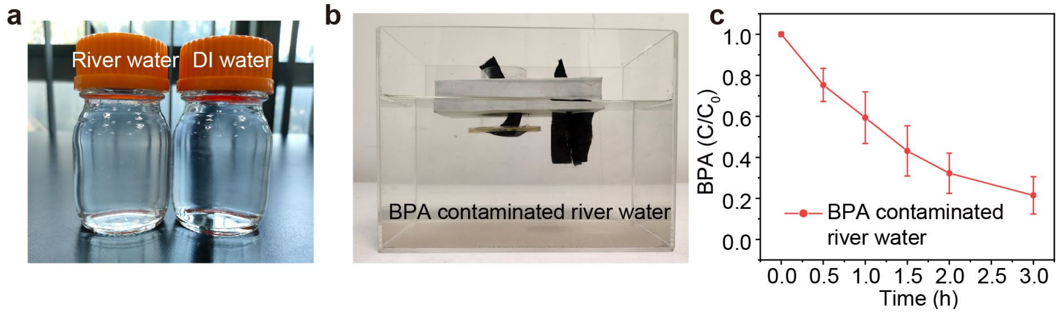

Supplementary Figure 30 | The catalytic performance of the device in the river water. a, Photographs of the river water and deionized water samples. **b**, Photograph of the floating device set-up placed on the water tank with 2 L of BPA-contaminated river water. **c**, Monitoring of the change in the corresponding BPA concentration within the tank. Conditions: BPA, 5 μM, 2 L; PDS, 10 mM, 40 mL. Experiments were conducted in triplicate, and the error bars represent the arithmetic mean ± standard deviation.

To explore the performance of our device towards real surface water, we took water sample directly from the Yuhangtang River, located at Zhejiang University's Zijingang campus in Hangzhou, Zhejiang province, China (Supplementary Figure 30a). The collected river water, augmented with a BPA solution, was utilized to directly evaluate the treatment efficacy employing our floating device (Supplementary Figure 30b). As depicted in Supplementary Figure 30c, it showed an obvious removal of BPA in the real river water.

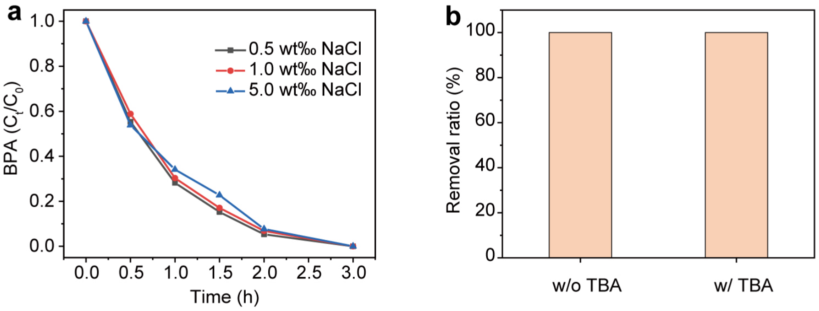

Supplementary Figure 31 | The influence of chloride ions. a, Measurement of the degradation curve of BPA under different concentration of NaCl. Conditions: BPA: 5 µM, PDS: 10 mM. **b**, The removal performance of BPA with/without the presence of *tert*-butyl alcohol. Conditions: BPA: 5 µM, PDS: 10 mM, NaCl: 0.5 wt‰, *tert*-butyl alcohol (TBA): 0.5 mM.

The effect of concentration of NaCl in the tank on the degradation of BPA was investigated in Supplementary Figure 31, suggesting the degradation rate of BPA did not change significantly with the increased NaCl concentration in the tank.

To explore the possibility of generation of Cl· radical in our system, *tert*-butyl alcohol (TBA, a typical scavenger for Cl·, reaction rate with Cl·: 3-19 × 10^8 M^{-1} s^{-1}) was added during the degradation experiment[9-11]. As unveiled by Supplementary Figure 31b, addition of TBA didn't cause obvious change on the BPA degradation performance, thus we can conclude that Cl· didn't participate in our reaction.

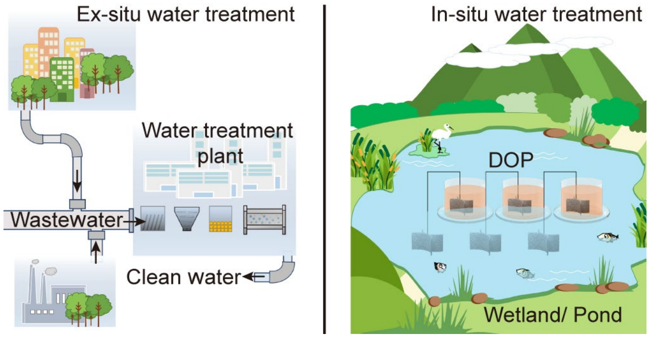

Supplementary Figure 32. Schematic illustration comparing the ex-situ water treatment of bio-refractory organic contaminants with centralized water treatment plants, and the potential of using our DOP device for in-situ treatment of contaminated surface streams such as river, lakes, wetlands and ponds.

Supplementary Table 1. The HPLC analysis conditions for different substrates.

Substrates	Flow rate (mL min^{-1})	CH_3OH (vol.%)	H_2O (vol.%)	λ (nm)
Bisphenol A	1.0	70.0	30.0	230.0
p-chlorophenol	1.0	60.0	40.0	225
2,4-dichlorophenol	1.0	70.0	30.0	295
Phenol	1.0	50.0	50.0	270
p-nitrophenol	1.0	70	30	280

Supplementary Table 2. Surface porosity and elemental composition of Cu-N-C-n

Samples	SSA (m^2 g^{-1})	Pore volume (cm^3 g^{-1})	Pore size (nm)	Atomic percentage (at%)			
				C 1s	N 1s	O 1s	Cu 2p
Cu-N-C-9	146.8265	0.424243	14.8459	86.69	9.06	3.63	0.61
Cu-N-C-8	199.5929	0.492561	13.9858	78.90	15.41	4.04	1.66
Cu-N-C-7	129.3947	0.428817	14.0285	67.94	25.58	4.49	1.99
Cu-N-C-6	87.7078	0.379587	16.6325	60.81	31.52	4.73	2.965

Supplementary Table 3. EXAFS fitting parameters at the Cu K-edge for various samples (S_0^2=0.87)

	Shell	CN	Content	R(Å)	σ^2	ΔE_0	R factor
Cu foil	Cu-Cu	12	-	2.54±0.01	0.0089	4.1±0.6	0.0029
Sample	$Cu\text{-}N_2$	2	0.3±0.1	1.79±0.03	0.0015	6.8±1.9	0.0162
	$Cu\text{-}N_4$	4	0.7±0.1	1.96±0.02	0.0047		

Note: *CN*: coordination numbers; *R*: bond distance; σ^2: Debye-Waller factors; ΔE_0: the inner potential correction; *R* factor: goodness of fit. S_0^2 was set to 0.87, according to the experimental EXAFS fit of Cu foil reference by fixing CN as the known crystallographic value.

Supplementary Table 4. Comparison of the catalytic performance of Cu-N-C-8 with the reported literatures

Catalyst	Oxidant dosage	Pollutant (mg L^{-1})	Removal efficiency	*k* value (min^{-1})	TOF (L g^{-1} min^{-1})	Reference
**Cu-N-C-8 (0.15 g L^{-1})**	**PDS (1 mM)**	**BPA (0.1 mM)**	**100% (5 min)**	**1.777**	**11.85**	**This work**
N-BC900 (0.2 g L^{-1})	PDS (2 mM)	BPA (88 μM)	100% (20 min)	0.214	1.07	Ref. 12
CNF3 (0.10 g L^{-1})	PMS (1 mM)	4-CP (0.1 mM)	100% (20 min)	0.254	2.54	Ref. 13
Fe-SACs (0.2 g L^{-1})	PMS (1.3 mM)	BPA (0.11 mM)	88% (30 min)	0.104	0.52	Ref. 14
$Fe_{0.15}Mn_{0.85}O_2$ (0.04 g L^{-1})	PMS (0.5 mM)	BPA (5 μM)	100% (21 min)	~0.23	~5.75	Ref. 15
CuO (0.2 g L^{-1})	PDS (40 μM)	2,4-DCP (5 μM)	100% (40 min)	0.062	0.31	Ref. 16
NBC-4 (1.0 g L^{-1})	PDS (1 mM)	SDZ (10 μM)	96.2% (60 min)	0.0798	0.0798	Ref. 17
Cu-N_4/C-B (0.1 g L^{-1})	PMS (0.65 mM)	BPA (88 μM)	98% (5 min)	0.56	5.6	Ref. 18
PSS-800 (0.2 g L^{-1})	PDS (1.85 mM)	2,4-DCP (0.62 mM)	100% (60 min)	0.0606	0.303	Ref. 19
Cu_{SA}-NC (0.04 g L^{-1})	PDS (0.5 mM)	2,4-DCP (0.1 mM)	100% (30 min)	0.284	7.1	Ref. 20
$ZnFeMnO_4$ (0.1 g L^{-1})	PMS (0.16 mM)	BPA (40 μM)	100% (15 min)	0.43	4.3	Ref. 21
Annealed CNT (0.1 g L^{-1})	PDS (1 mM)	Phenol (0.1 mM)	100% (30 min)	0.198	1.98	Ref. 22

Note: The turnover frequency (TOF) was calculated through dividing the reaction rate of pollutant degradation by the catalyst concentration.

Supplementary Table 5. The respective calculation of the fabrication cost for different electrode

Electrode		Resource	Unit Price[1,2] ($)	Amount/size used for one electrode	Partial cost for one electrode ($)
Cu-N-C@GF	Material	Copper (II) acetate monohydrate	4.75 kg^{-1}	5.08 mg	2.41×10^{-5}
		L-glutamic acid	1.40 kg^{-1}	1.87 mg	2.62×10^{-6}
		1,3,5-benzenetricarboxylic acid	4.89 kg^{-1}	2.81 mg	1.37×10^{-5}
		Dicyandiamide	2.79 kg^{-1}	41.15 mg	1.15×10^{-4}
		**Cu-N-C**	**43.1** kg^{-1}	**3.60 mg**	$\mathbf{1.55\times10^{-4}}$
		Nafion solution	1.61 mL^{-1}	3.60 μL	5.80×10^{-3}
		Graphite felts	21.0 m^{-2}	6.00 cm^2	1.26×10^{-2}
	Electricity	Fabrication of Cu-N-C	0.140 kWh^{-1}	3.56×10^{-2} kWh	4.98×10^{-3}
		Fabrication of Cu-N-C@GF	0.140 kWh^{-1}	0.363 kWh	5.08×10^{-2}
**Estimated cost for one Cu-N-C@GF electrode**					**~ $ 0.0744**
CNTs@GF	Material	**CNTs**	**100.0** kg^{-1}	**3.60 mg**	$\mathbf{3.60\times10^{-4}}$
		Nafion solution	1.61 mL^{-1}	3.60 μL	5.80×10^{-3}
		Graphite felts	21.0 m^{-2}	6.00 cm^2	1.26×10^{-2}
	Electricity	Fabrication of CNTs	-	-	-
		Fabrication of CNTs@GF	0.140 kWh^{-1}	0.363 kWh	5.08×10^{-2}
**Estimated cost for one CNTs@GF electrode**					**~ $ 0.0696**
CuO@GF	Material	**CuO**	**11.3** kg^{-1}	**3.60 mg**	$\mathbf{4.08\times10^{-5}}$
		Nafion solution	1.61 mL^{-1}	3.60 μL	5.80×10^{-3}
		Graphite felts	21.0 m^{-2}	6.00 cm^2	1.26×10^{-2}
	Electricity	Fabrication of CuO	-	-	-
		Fabrication of CuO@GF	0.140 kWh^{-1}	0.363 kWh	5.08×10^{-2}
**Estimated cost for one CuO@GF electrode**					**~ $ 0.0692**

Note: [1]Prices of the chemicals were determined based on the average prices on website. [2]Price of the electricity was determined according to the average electricity price in China.

Supplementary Table 6. Comparison of estimated cost for treating 2500 m^3 amount of impaired water with different techniques

	Construction cost for 2500 m^3 water treatment ability	Operation cost			Characteristics of operation	Total Cost/2500 m^3 impaired water
		Electricity consumption /m^3 impaired water	Cost depletion	Chemical usage/2500 m^3 impaired water		
Our floating device with decoupled oxidation process (DOP)	Electrodes + Floating set ~$ 5.9 per set $ 5.9 ×100 sets = **$ 590** for treating 2500 m^3 impaired water at site[a]	No power consumption	Cu-N-C@GF electrode ~$ 0.73 per set $ 0.73 ×100 sets ÷10 = **$ 7.3**[d]	~2.7 kg of PDS, ~$ 2.7[e]	**in-situ and "self-responsive"**	**~$ 600**
Conventional AOP water treatment station	Water tank+ Pump + Pipeline + Electric generator + Treatment system **>$ 10,000**[b]	~3.12 $kWhm^{-3}$ × ~$ 0.36 kWh^{-1} × 2500 m^3=**$ 2808**[c]	No loss of equipment in short term	~2.7 kg of PDS, ~$ 2.7[f]	**off-site, need monitoring on the occurrence of pollutants**	**>$ 13,000**

Note: a. [1]The set used here is the enlarged set which can contain 1 L 10 mM PDS solution. [2]The price was calculated based on the materials involved in the device fabrication, including the graphite felts, chemicals for catalyst synthesis, cylinder tube, copper wire and etc.
b. Prices of the equipment were determined based on the average prices on website.
c. The average $kWhm^{-3}$ was calculated based on the average amount given in reference[23].
d. According to Supplementary Figure 21 above, our catalytic electrode showed stable performance during a ten cycled degradation, thus the loss coefficient of the catalytic electrode was estimated to be 0.1.
e. The consumed PDS amount and price was calculated based on the experiment on Supplementary Figure 5 and price searched on website.
f. We estimated the PDS amount in conventional AOP as the same amount in our decoupled AOP, however, in dealing with trace micropollutants, the conventional AOPs always require oxidants with an overdose amount, due to the radical scavenging effects[24,25].

The conventional off-site approaches always need water pumps to circulate water for treatments, resulting in considerable energy costs (e.g. pumping 2500 m^3 of water may necessitate 7800 kWh of electricity, as shown in Supplementary Table 6). These methods are difficult to be applied at remote areas where power supplies and infrastructures are barely implemented, such as wild wetlands, isolated islands, and rural regions. By contrast, our in-situ floating approach can be directly deployed in bulk waterbodies, requiring no external water pumps and electric consumption. It makes this approach potentially promising for low-cost decentralized water treatments in remote and pollution-scattered areas. It offers a viable solution for pollution control in area-source

pollution scenarios, where the large volume of polluted water cannot be pumped out due to logistical constraints.

Supplementary Table 7. Parameters of the modular device and water tank involved in the 6-day continuous treatment.

	Diameter (m)	Occupied Area (m^2)	Volume (L)
Modular device	0.04	0.005	0.04
Tank with impaired water	1.5	7.065	200
Impaired water /device	37.5	**1413**	**5000**

Supplementary References

1. Li, F. *et al.* Boosting oxygen reduction catalysis with abundant copper single atom active sites. *Energy Environ. Sci.* **11**, 2263-2269 (2018).

2. Gascon, J., Aguado, S. & Kapteijn, F. Manufacture of dense coatings of $Cu_3(BTC)_2$ (HKUST-1) on α-alumina. *Microporous Mesoporous Mater.* **113**, 132-138 (2008).

3. Li, X. *et al.* CoN_1O_2 Single-Atom Catalyst for Efficient Peroxymonosulfate Activation and Selective Cobalt(IV)=O Generation. *Angew. Chem. Int. Ed.* **62**, e202303267 (2023).

4. Mi, X. *et al.* Almost 100 % Peroxymonosulfate Conversion to Singlet Oxygen on Single-Atom CoN_{2+2} Sites. *Angew. Chem. Int. Ed.* **60**, 4588-4593 (2021).

5. Thorum, M. S., Hankett, J. M. & Gewirth, A. A. Poisoning the Oxygen Reduction Reaction on Carbon-Supported Fe and Cu Electrocatalysts: Evidence for Metal-Centered Activity. *J. Phys. Chem. Lett.* **2**, 295-298 (2011).

6. Tan, W. *et al.* Peroxymonosulfate activated with waste battery-based Mn-Fe oxides for pollutant removal: Electron transfer mechanism, selective oxidation and LFER analysis. *Chem. Eng. J.* **394**, 124864 (2020).

7. Shao, P. *et al.* Potential Difference Driving Electron Transfer via Defective Carbon Nanotubes toward Selective Oxidation of Organic Micropollutants. *Environ. Sci. Technol.* **54**, 8464-8472 (2020).

8. Ren, W. *et al.* Activation of Peroxydisulfate on Carbon Nanotubes: Electron-Transfer Mechanism. *Environ. Sci. Technol.* **53**, 14595-14603 (2019).

9. Song, D. *et al.* Degradation of Perfluorooctanoic Acid by Chlorine Radical Triggered Electrochemical Oxidation System. *Environ. Sci. Technol.* **57**, 9416-9425 (2023).

10. Barazesh, J. M., Prasse, C. & Sedlak, D. L. Electrochemical Transformation of Trace Organic Contaminants in the Presence of Halide and Carbonate Ions. *Environ. Sci. Technol.* **50**, 10143-10152 (2016).

11. Kong, X. *et al.* Degradation of atrazine by UV/chlorine: Efficiency, influencing factors, and products. *Water Res.* **90**, 15-23 (2016).

12. Zhu, S. *et al.* Catalytic Removal of Aqueous Contaminants on N-Doped Graphitic Biochars: Inherent Roles of Adsorption and Nonradical Mechanisms. *Environ. Sci. Technol.* **52**, 8649-8658 (2018).

13. Li, H., Shan, C. & Pan, B. Fe(III)-Doped g-C_3N_4 Mediated Peroxymonosulfate Activation for Selective Degradation of Phenolic Compounds via High-Valent Iron-Oxo Species. *Environ. Sci. Technol.* **52**, 2197-2205 (2018).

14. Gao, Y. *et al.* Activity Trends and Mechanisms in Peroxymonosulfate-Assisted Catalytic Production of Singlet Oxygen over Atomic Metal-N-C Catalysts. *Angew. Chem. Int. Ed.* **60**, 22513-22521 (2021).

15. Huang, K. Z. & Zhang, H. Direct Electron-Transfer-Based Peroxymonosulfate Activation by Iron-Doped Manganese Oxide (δ-MnO2) and the Development of Galvanic Oxidation Processes (GOPs). *Environ. Sci. Technol.* **53**, 12610-12620 (2019).

16. Zhang, T. *et al.* Efficient Peroxydisulfate Activation Process Not Relying on Sulfate Radical Generation for Water Pollutant Degradation. *Environ. Sci. Technol.* **48**, 5868-5875 (2014).

17. Wang, H. *et al.* Edge-nitrogenated biochar for efficient peroxydisulfate activation: An electron transfer mechanism. *Water Res.* **160**, 405-414 (2019).

18. Zhou, X. *et al.* Identification of Fenton-like active Cu sites by heteroatom modulation of electronic density. *Proc. Natl. Acad. Sci.* **119**, e2119492119 (2022).

19. Yu, J. *et al.* Hierarchical porous biochar from shrimp shell for persulfate activation: A two-electron transfer path and key impact factors. *Appl. Catal. B Environ.* **260**, 118160 (2020).

20. Li, F. *et al.* Origin of the Excellent Activity and Selectivity of a Single-Atom Copper Catalyst with Unsaturated Cu-N_2 Sites via Peroxydisulfate Activation: Cu(III) as a Dominant Oxidizing Species. *Environ. Sci. Technol.* **56**, 8765-8775 (2022).

21. Pei, D.-N. *et al.* In situ organic Fenton-like catalysis triggered by anodic polymeric intermediates for electrochemical water purification. *Proc. Natl. Acad. Sci.* **117**, 30966-30972 (2020).

22. Ren, W. *et al.* Insights into the Electron-Transfer Regime of Peroxydisulfate Activation on Carbon Nanotubes: The Role of Oxygen Functional Groups. *Environ. Sci. Technol.* **54**, 1267-1275 (2020).

23. Żyłka, R., Karolinczak, B. & Dąbrowski, W. Structure and indicators of electric energy consumption in dairy wastewater treatment plant. *Sci. Total Environ.* **782**, 146599 (2021).

24. Alsbaiee, A. *et al.* Rapid removal of organic micropollutants from water by a porous β-cyclodextrin polymer. *Nature* **529**, 190-194 (2016).

25. Yang, Z. *et al.* Toward Selective Oxidation of Contaminants in Aqueous Systems. *Environ. Sci. Technol.* **55**, 14494-14514 (2021).
