# Supplementary material for: Decoupled oxidation process enabled by atomically dispersed copper electrodes for in-situ chemical water treatment
Source: Nat Commun. 2024 Feb 8;15:1186. doi: 10.1038/s41467-024-45481-y (PMC10853265; doi:10.1038/s41467-024-45481-y)
Supplement: Supplementary file 3 — Reporting Summary [file 41467_2024_45481_MOESM3_ESM.pdf]

Corresponding author(s): Juan Wang

Last updated by author(s): Jan 20, 2014

Reporting Summary

Nature Portfolio wishes to improve the reproducibility of the work that we publish. This form provides structure for consistency and transparency in reporting. For further information on Nature Portfolio policies, see our Editorial Policies and the Editorial Policy Checklist.

Statistics

For all statistical analyses, confirm that the following items are present in the figure legend, table legend, main text, or Methods section.

n/a	Confirmed	
☐	☒	The exact sample size (*n*) for each experimental group/condition, given as a discrete number and unit of measurement
☐	☒	A statement on whether measurements were taken from distinct samples or whether the same sample was measured repeatedly
☐	☒	The statistical test(s) used AND whether they are one- or two-sided<br>*Only common tests should be described solely by name; describe more complex techniques in the Methods section.*
☒	☐	A description of all covariates tested
☒	☐	A description of any assumptions or corrections, such as tests of normality and adjustment for multiple comparisons
☐	☒	A full description of the statistical parameters including central tendency (e.g. means) or other basic estimates (e.g. regression coefficient) AND variation (e.g. standard deviation) or associated estimates of uncertainty (e.g. confidence intervals)
☐	☒	For null hypothesis testing, the test statistic (e.g. *F*, *t*, *r*) with confidence intervals, effect sizes, degrees of freedom and *P* value noted<br>*Give P values as exact values whenever suitable.*
☒	☐	For Bayesian analysis, information on the choice of priors and Markov chain Monte Carlo settings
☒	☐	For hierarchical and complex designs, identification of the appropriate level for tests and full reporting of outcomes
☒	☐	Estimates of effect sizes (e.g. Cohen's *d*, Pearson's *r*), indicating how they were calculated

Our web collection on statistics for biologists contains articles on many of the points above.

Software and code

Policy information about availability of computer code

Data collection	n/a
Data analysis	n/a

For manuscripts utilizing custom algorithms or software that are central to the research but not yet described in published literature, software must be made available to editors and reviewers. We strongly encourage code deposition in a community repository (e.g. GitHub). See the Nature Portfolio guidelines for submitting code & software for further information.

Data

Policy information about availability of data

All manuscripts must include a data availability statement. This statement should provide the following information, where applicable:

- Accession codes, unique identifiers, or web links for publicly available datasets
- A description of any restrictions on data availability
- For clinical datasets or third party data, please ensure that the statement adheres to our policy

The data that support the findings of this study are available from the corresponding author upon reasonable request.

Research involving human participants, their data, or biological material

Policy information about studies with human participants or human data. See also policy information about sex, gender (identity/presentation), and sexual orientation and race, ethnicity and racism.

Reporting on sex and gender	n/a
Reporting on race, ethnicity, or other socially relevant groupings	n/a
Population characteristics	n/a
Recruitment	n/a
Ethics oversight	n/a

Note that full information on the approval of the study protocol must also be provided in the manuscript.

Field-specific reporting

Please select the one below that is the best fit for your research. If you are not sure, read the appropriate sections before making your selection.

☐ Life sciences ☐ Behavioural & social sciences ☒ Ecological, evolutionary & environmental sciences

For a reference copy of the document with all sections, see nature.com/documents/nr-reporting-summary-flat.pdf

Ecological, evolutionary & environmental sciences study design

All studies must disclose on these points even when the disclosure is negative.

Study description	We designed a novel decoupled AOP approach for in-situ water treatment. Moreover, the degradation of organic pollutants such as bisphenol A by this approach in water and the lethal effects of this method on organisms in water such as zebrafish were studied. All the experiments were repeated for three times for each group.
Research sample	About the degradation of organic pollutants, we chose the reaction volume of 40 mL, 2 L and 200 L, respectively, to illustrate efficiency of our devices in different application scenarios. And the reaction volume chosen based on published reports. Zebrafish are commonly used in laboratory animals. Therefore, to demonstrate that the decoupled AOP approach in this study could reduce the harm of persulfate to aquatic fish, we used commercially farmed zebrafish as samples. The datasets are all generated during and/or analysed during the current study.
Sampling strategy	Samples were selected by random sampling, and the sample size was determined according to the suitable breeding density of zebrafish. As the experimental fish tank was suitable for holding 4 L water, the amount of zebrafish in each group of experiments was finally determined to be 20.
Data collection	During the each reaction, the data was recorded and the number of dead zebrafish was counted by Ziwei Yu at scheduled time intervals.
Timing and spatial scale	There was no interruption between the beginning of each experiment and the end of the experiment period.
Data exclusions	No data were excluded from the analyses.
Reproducibility	All attempts to repeat the experiment were successful.
Randomization	Samples were selected by random sampling.
Blinding	The zebrafish in the study didn't know if their environment was bad for survival.

Did the study involve field work? ☐ Yes ☒ No

Reporting for specific materials, systems and methods

We require information from authors about some types of materials, experimental systems and methods used in many studies. Here, indicate whether each material, system or method listed is relevant to your study. If you are not sure if a list item applies to your research, read the appropriate section before selecting a response.

Materials & experimental systems

n/a	Involved in the study
x	Antibodies
x	Eukaryotic cell lines
x	Palaeontology and archaeology
	x Animals and other organisms
x	Clinical data
x	Dual use research of concern
x	Plants

Methods

n/a	Involved in the study
x	ChIP-seq
x	Flow cytometry
x	MRI-based neuroimaging

Animals and other research organisms

Policy information about studies involving animals; ARRIVE guidelines recommended for reporting animal research, and Sex and Gender in Research

Laboratory animals	This study involved adult zebrafish (about 3 months).
Wild animals	n/a
Reporting on sex	Findings were not limited by sex.
Field-collected samples	The experiments were all done in the room with the room temperature of about 25 degrees Celsius.
Ethics oversight	We have followed the ethical guidelines for animal research provided by Nature Portfolio.

Note that full information on the approval of the study protocol must also be provided in the manuscript.

Plants

Seed stocks	n/a
Novel plant genotypes	n/a
Authentication	n/a
